# Pre-diagnosis blood glucose and prognosis in women with breast cancer

**DOI:** 10.1186/s40170-016-0147-7

**Published:** 2016-04-06

**Authors:** Behjatolah Monzavi-Karbassi, Rhonda Gentry, Varinder Kaur, Eric R. Siegel, Fariba Jousheghany, Srikanth Medarametla, Barbara J. Fuhrman, A. Mazin Safar, Laura F. Hutchins, Thomas Kieber-Emmons

**Affiliations:** Winthrop P. Rockefeller Cancer Institute, University of Arkansas for Medical Sciences, Little Rock, AR 72205 USA; Department of Pathology, University of Arkansas for Medical Sciences, 4301 West Markham St., Slot #824, Little Rock, AR 72205 USA; Division of Hematology/Oncology, University of Arkansas for Medical Sciences, Little Rock, AR 72205 USA; Department of Biostatistics, University of Arkansas for Medical Sciences, Little Rock, AR 72205 USA; Department of Epidemiology, University of Arkansas for Medical Sciences, Little Rock, AR 72205 USA

**Keywords:** Breast cancer, Blood glucose, Patient survival

## Abstract

**Background:**

The effect of moderately elevated blood glucose levels among non-diabetic subjects on cancer prognosis is not well described. The goal of this study was to examine the association of elevated random blood glucose (RBG) levels in non-diabetic breast cancer patients with overall survival (OS) and time to tumor recurrence (TTR).

**Results:**

Forty-nine deaths and 32 recurrences occurred among 148 eligible study subjects during 855.44 person-years of follow-up, with median follow-up of 5.97 years. We observed that patients with elevated RBG levels experienced significantly shorter OS (hazard ratio [HR], 3.01; 95 % confidence interval [CI] (1.70–5.33); *P* < 0.001) and shorter TTR (HR, 2.08; CI (1.04–4.16); *P* = 0.04) as compared to patients with non-elevated RBG levels. After controlling for tumor grade, tumor stage, race, and BMI, elevated RBG continued to display high and statistically significant association with shorter OS (HR, 3.50; CI (1.87–6.54); *P <* 0.001). Adjustment for age, race, and BMI strengthened HR of RBG for TTR. The association of RGB with TTR lost its borderline statistical significance upon controlling for both tumor grade and stage.

**Conclusions:**

The data suggest that elevated blood glucose is associated with poor prognosis of breast cancer patients. Given the potential clinical implication, these findings warrant further investigation.

## Background

Numerous epidemiologic studies indicate that type 2 diabetes is associated with an increased risk of breast cancer [1–6]. Several large studies that compared cancer-related mortality between subjects with and without diabetes suggested an association between increased mortality and diabetes for breast cancer [1, 7–9]. However, the contribution of other factors including the delay in diagnosis, lower use of effective adjuvant therapies, diabetes-prescribed drug use, and diabetes-related comorbidities on observed association between diabetes and breast cancer is not clearly addressed [9–20]. To manage the unpredictable impact of the confounding factors on the observational studies, Boyle et al. [21] investigated the association of elevated blood glucose and breast cancer risk in women without diabetes and reported a small increase in breast cancer risk in a meta-analysis study. With regard to the published data, studying breast cancer outcome among non-diabetic subjects with elevated blood glucose levels is clearly needed.

Random blood glucose (RBG) values ≥120 mg/dL have been shown to have 90–92 % specificity for detection of any glucose intolerance that include patients with pre-diabetes and undiagnosed diabetes [22, 23]. We adopted RBG values of 120 mg/dL or higher to be an indication of disorders in glucose metabolism [22, 24], and performed a retrospective cohort study to evaluate the relationship between elevated blood glucose levels and survival of non-diabetic breast cancer subjects. We observed that elevated RBG levels correlate with poor prognosis in breast cancer patients independently of age, tumor characteristics, race, and BMI.

## Methods

### Study design and characteristics of patient population

This was a retrospective chart-review cohort study that was performed according to a protocol approved by the UAMS Institutional Review Board (IRB). The UAMS IRB determined the study to met the criteria for exempt status per 45 CFR 46, meaning there was no requirement to obtain informed consent from the study subjects. A cohort was formed by including subjects diagnosed with breast cancer from 1995 to 2000. African-American (AA) and European-American (EA) women who were ≥18 years of age at diagnosis, and who had at least one RBG measurement from 1 year before cancer diagnosis to the time of diagnosis, were included. Patients with a diagnosis of diabetes at any time before cancer diagnosis to the last follow-up date were excluded. Patients with documented disease stages I, II, and III were included, whereas patients with stage IV (metastatic) disease were excluded. A total of 148 patients were identified, all of whom were treated at the hospital of University Arkansas for Medical Sciences (UAMS). The dates of diagnosis, tumor recurrence, and last follow-up, vital status, and disease status at the last follow-up, age, race, tumor stage and grade were provided by the UAMS tumor registry. RBG, height, and weight were extracted from the medical-record charts.

### Outcome and predictor variables

There were two measures of outcome: overall survival (time to death by any reason with censoring occurring at last contact when patient was still alive) and time to tumor recurrence (with censoring at death or last contact). Measured RBG levels were classified as “high” or “elevated” if ≥120 mg/dL versus “low” or “non-elevated” if <120 mg/dL, and this dichotomous classification was used as the main predictor variable. The RBG measurements evaluated in this study included data collected within a 1-year period prior to the diagnosis, and were averaged together if multiple numbers were recorded to reach a single measurement for each patient. Height, weight, age, and race were recorded at the time of diagnosis. BMI (trichotomized as ≥30, <30, and unknown), race (AA vs. EA), age (as a continuous variable), tumor grade (as a categorical variable), and stage (as a categorical variable) were used as covariates.

### Statistical analysis

Survival and time to tumor recurrence in the high and low RBG groups were visualized using Kaplan-Meier curves and compared for differences with the log-rank test. After confirming the validity of the proportional-hazards assumption, outcomes were analyzed for covariate associations using univariate and multivariate Cox-regression analyses. In order to avoid overfitting the multivariate Cox models, we required every model to have a minimum of 10 observed events per covariate [25]. This meant that the maximum number of predictor variables in a multivariate Cox model was five for analysis of overall survival (OS) and three for analysis of time to tumor recurrence (TTR). Hazard ratios (HRs) with 95 % confidence intervals were calculated for each covariate. All hypothesis tests were two-sided; all *P* values were reported numerically, and evaluated using an alpha = 0.05 significance level. IBM SPSS Statistics Version 22 (IBM Corp., Armonk, NY) software was used for statistical analyses.

## Results

### Characteristics of patient population

Forty-nine deaths and 32 recurrences occurred among 148 eligible study subjects during 855.44 person-years of follow-up, with median follow-up of 5.97 years. Distributions of selected variables in studied population are summarized in Table 1. Of the total number of the patients, 120 (81 %) were EA and 28 (19 %) were AA women. Most patients (123, 83 %) were diagnosed with stage II breast cancer, although 20 (14 %) and 5 (3 %) of total eligible subjects were respectively diagnosed with stages I and III disease. Sixty-eight patients (46 %) were diagnosed with pathological grade III tumors versus 54 (36 %) with grade II and 26 (18 %) with grade I. Age was distributed with a minimum, median, and maximum age of 28, 53, and 89 years, respectively.

**Table 1:** Characterization of the studied population

Characteristics	RBG < 120	RBG ≥ 120	Total	*P* value
	No. (%)	No. (%)	No. (%)	
All patients	95 (64 %)	53 (36 %)	148 (100 %)	
Age				0.02
Min	29	28	28	
Max	89	85	89	
Mean (SD)	53.3 (13.3)	57.4 (12.9)	54.80 (13.3)	
Median	50	56	53.0	
<50	45 (47 %)	15 (28 %)	60 (41 %)	
≥50	50 (53 %)	38 (72 %)	88 (59 %)	
Tumor grade				0.14
Grade I	21 (22 %)	5 (9 %)	26 (18 %)	
Grade II	34 (36 %)	20 (38 %)	54 (36 %)	
Grade III	40 (42 %)	28 (53 %)	68 (46 %)	
Tumor stage				0.19
Stage I	16 (17 %)	4 (8 %)	20 (14 %)	
Stage II	75 (79 %)	48 (91 %)	123 (83 %)	
Stage III	4 (4 %)	1 (2 %)	5 (3 %)	
Race				0.05
EA	82 (86 %)	38 (72 %)	120 (81 %)	
AA	13 (14 %)	15 (28 %)	28 (19 %)	
BMI				0.1
Min	17	21	17	
Max	54	43	54	
Mean (SD)	28.5 (6.8)	30.3 (6.3)	29.1 (6.6)	
Median	27	29	27	
<30	61 (64 %)	25 (47 %)	49 (33 %)	
≥30	28 (29 %)	21 (40 %)	86 (58 %)	
Unknown	6 (6 %)	7 (13 %)	13 (9 %)	
Death	20 (41 %)	29 (59 %)	49 (100 %)	
Recurrence	16 (50 %)	16 (50 %)	32 (100 %)	
Median follow-up (years)	6.27	5.14	5.97	

### Elevated RBG correlates with poor prognosis and shorter time to tumor recurrence

**Fig. 1:**
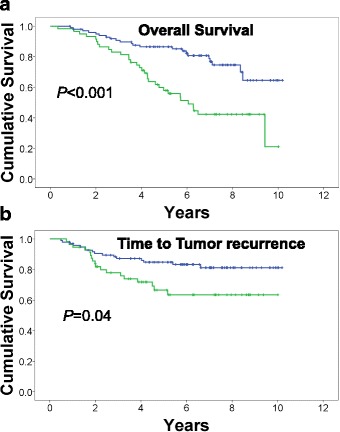
Kaplan-Meier analysis of RBG and patients’ survival. High RBG levels are associated with shorter overall survival (**a**) and shorter time to tumor recurrence (**b**). RGB values of ≥120 mg/dL were considered high. *P* values were estimated using log-rank test. *Green*, RBG ≥ 120; *blue*, RBG < 120. *Plus signs* indicate censoring

We adopted RBG values of 120 mg/dL or higher to be an indication of disorders in carbohydrate metabolism [22, 24]. An RBG cutoff value of 120 mg/dL has been shown to have 90–92 % specificity in the detection of any glucose intolerance, which includes patients with pre-diabetes and undiagnosed diabetes [22, 23]. We performed survival analysis to evaluate the association of high RBG, a likely representative of pre-diabetes, with patients’ survival and time to tumor recurrence. Patients with elevated RBG had significantly shorter overall survival and time to tumor recurrence (Fig. 1). Subjects in elevated RBG group had a median ± SE of 6.05 ± 0.65 for OS. Median OS was not reached for subjects with low RBG. Neither of the study subpopulations reached median TTR.

### Association of RBG with survival persists after controlling for tumor characteristics, age, BMI, and race

Tumor grade and tumor stage are the common prognostic factors that correlate with recurrence and survival endpoints. Race also affects survival, since AA breast cancer patients experience shorter survival time than their EA counterparts [26]. RBG levels may associate with BMI. Therefore, the association of RBG with both OS and TTR was evaluated after controlling for age, tumor grade, tumor stage, BMI, and race (Table 2). RBG level of ≥120 mg/dL was associated with a shorter OS (hazard ratio [HR] = 3.01; 95 % confidence interval [CI], (1.70–5.33); *P <* 0.001), and a shorter time to tumor recurrence (HR, 2.08; CI (1.04–4.16), *P =* 0.039). In univariate analysis, none of the other variables showed significant association with OS, and only age displayed a significant association with TTR (HR, 0.96; CI (0.94–0.99), *P =* 0.01).

**Table 2:** Hazard ratios for the association between RBG (≥120 vs. <120) and breast cancer outcomes

Overall survival	HR (95 % CI)	*P *value
Crude	3.01 (1.70–5.33)	<0.001
Adjusted for age	2.99 (1.68–5.33)	<0.001
Adjusted for age group (≥50 vs. <50)	3.07 (1.72–5.48)	<0.001
Adjusted for grade	3.08 (1.71–5.57)	<0.001
Adjusted for stage	3.09 (1.73–5.53)	<0.001
Adjusted for age, tumor grade and tumor stage	3.19 (1.74–5.86)	<0.001
Adjusted for age, tumor grade, tumor stage, and race	3.24 (1.75–5.99)	<0.001
Adjusted for age group (≥50 vs. <50), tumor grade, tumor stage, and race	3.40 (1.83–6.33)	<0.001
Adjusted for BMI	3.24 (1.78–5.89)	<0.001
Adjusted for tumor grade, tumor stage, race, and BMI	3.50 (1.87–6.54)	<0.001
Time to tumor recurrence^a^		
Crude	2.08 (1.04–4.16)	0.039
Adjusted for age	2.59 (1.27–5.26)	0.009
Adjusted for age group (≥50 vs. <50)	2.65 (1.29–5.44)	0.008
Adjusted for grade	2.06 (1.01–4.18)	0.046
Adjusted for stage	2.01 (1.00–4.04)	0.051
Adjusted for grade and stage	2.00 (0.97–4.09)	0.059
Adjusted for race	2.17 (1.08–4.38)	0.030
Adjusted for BMI	2.37 (1.16–4.83)	0.017
Adjusted for BMI and grade	2.32 (1.13–4.73)	0.021
Adjusted for BMI and stage	2.32 (1.13–4.76)	0.022

^a^Five out of 148 (3.4 %) subjects who never became disease free were removed from TTR analyses

Controlling for tumor grade, tumor stage, or age did not appreciably change the HR value of RBG for OS (Table 2). However, adjustment for these three covariates combined led to a 6 % increase in the HR value of RBG for OS, from 3.01 to 3.19 (Table 2). The addition of race to this model (adjusting for age, tumor grade, tumor stage, and race) barely affected the associations of RBG with OS. Controlling for BMI improved association of RBG with OS (HR increased from 3.01 to 3.24, an 8 % increase). After adjusting for tumor grade, tumor stage, race, and BMI, the HR value of RBG for OS increased by 16 % (HR, 3.50; CI (1.87–6.54); *P* < 0.001). With respect to TTR, controlling for age led to a 25 % increase in the HR value of RBG compared to its value from univariate analysis (Table 2). Adjusting for both tumor grade and tumor stage slightly weakened RBG’s association with TTR, as the HR dropped from 2.08 before adjustment to 2.00 (a 4 % decrease) after adjustment, but the weakening was enough to render the HR statistically insignificant. Adjusting for BMI strengthened the association of RBG and TTR where HR increased to 2.37 from 2.08 (a 14 % increase). The HR of TTR with RBG showed consistent increases over the crude estimate after controlling for either BMI and grade or BMI and stage (Table 2). These data suggest that RBG levels in breast cancer patients correlate with patients’ survival and probably recurrence of the disease.

## Discussion

Our study revealed that breast cancer patients with elevated RBG levels have shorter OS and TTR. Epidemiological studies strongly suggest an association between high blood glucose and mortality in breast cancer patients [9]. In a study examining the general population, Saydah et al. [27] classified the subjects as having either diagnosed diabetes, undiagnosed diabetes, impaired glucose tolerance, or normal glucose tolerance, and went on to observe that individuals with impaired glucose tolerance had the highest adjusted relative hazard of cancer mortality compared to the reference group with normal glucose tolerance. Consistently, our data suggest that RBG, which is a non-fasting blood glucose measurement, has a significant association with breast cancer outcomes, particularly with OS.

Controlling for tumor grade, tumor stage, and age suggests that RBG can be considered as an independently significant factor that correlates with overall survival. Age displayed a strong confounding effect on the RBG association with TTR, such that controlling for age accentuated the RBG association by 25 %. On the other hand, the prognostic significance of RBG for TTR, which was already at borderline, crossed the border into insignificance upon controlling for tumor grade and stage. Strengthening of the HR of RBG for OS after adjusting for race or BMI suggests that these two covariates confound the association of RBG with patients’ survival. A similar phenomenon was observed regarding the association of RBG with TTR. Therefore, race and BMI should be considered as major confounders in the design and conduct of future studies.

A diabetic population is a heterogeneous group to be studied for cancer mortality for at least three reasons. First, clinical diagnosis of diabetes often leads to treatment with anti-diabetic medications that reduce blood glucose, thus reducing the impact of diabetes on the tumor, and potentially introducing a source of variability when a given anti-diabetes drug may directly affect the tumor [12, 18–20, 28]. Second, there may be disease-dependent variation in glucose and insulin levels in diabetics. Third, there is a complex set of relationships between diabetes and cancer, and an incomplete understanding of underlying biological mechanisms associating these two health issues. Similar factors as above may contribute to heterogeneity of the grouping and play well into low power of the analyses. The fact that we observed a strong association between RBG and survival may suggest that RBG screening may have significant clinical implications. Pre-diabetes is a condition that occurs when a person’s blood glucose levels are higher than normal, though not high enough for a diagnosis of diabetes. People with pre-diabetes develop insulin resistance and are at an increased risk of type 2 diabetes. Measuring RBG levels has been proposed as an easy-to-measure and reliable predictor of pre-diabetes and early diabetes [22, 24]. Screening for high RBG may pick patients with undiagnosed early diabetes or in the pre-diabetes stage. Therefore, lack of drug use and being early in the process of development of the disease makes such a group of patients a relatively homogenous one, allowing to reduce confounders and improve confidence levels compared to cancer studies on diabetics or obese populations.

High glucose levels may be accompanied with high insulin levels. Insulin enhances cell growth and promotes cell proliferation due to its mitogenic effects [29]. High levels of insulin were associated with distant recurrence and poor survival [30–32]. We were not able to separate the impact of blood glucose and insulin on the survival endpoints. The possibility that breast cancer further increased the levels of RBG cannot be excluded, which is a limitation of this study. Moreover, treatment for breast cancer may increase RBG further, and adversely affect survival [33]. For example, the glucocorticoid Dexamethasone, which is widely used to prevent side effects during chemotherapy, is associated with elevation of blood glucose [34]. The use of this drug may unevenly affect patients with elevated RGB than those with low RGB levels. We were not able to address this issue, and further studies are required to explore these possibilities.

The hormone-receptor details needed to identify basal-like disease were not available for this study, and, therefore, we were not able to rule in or out any contribution for the breast cancer subtypes in the observed association between survival and RBG. Therefore, we cannot exclude the possibility that differences in the prevalence of triple-negative cancers contributed to the poorer survival among patients with high blood glucose. Other limitations of this study are small sample size and lack of cancer-specific mortality data that potentially can affect the outcome of this study. Availability of pre-diagnosis blood sugar measurement, while could have increased homogeneity may have also introduced unknown factors in selection of the patient population. Therefore, these results should be interpreted with caution.

## Conclusions

This study suggests that hyperglycemia, a component of pre-diabetes and undiagnosed early diabetes, affects survival of breast cancer patients. Therefore, a routine measurement of blood glucose may potentially give guidance on mortality risk for breast cancer patients. As the prevalence of disorders in carbohydrate metabolism grows, the population of breast cancer patients with this disorder is also growing, and our study suggests that these patients tend to experience shorter survival than patients with normal blood glucose levels. Our findings could represent a great public health issue and therefore warrant further investigation in larger cohorts.

## Abbreviations

AA: African American
BMI: body mass index
CI: confidence interval
EA: European American
HR: hazard ratio
IRB: Institutional Review Board
OS: overall survival
RBG: random blood glucose
TTR: time to tumor recurrence
UAMS: University of Arkansas for Medical Sciences
